# Clinical Management of Self-Harming Children and Adolescents in the United Kingdom: A Student-Led Multicentre Audit

**DOI:** 10.1192/bjo.2025.10395

**Published:** 2025-06-20

**Authors:** Heather McAdam, Ruth Goh, Gloria Cheung, Felicity Allman, Samyak Pandey

**Affiliations:** 1 University of Glasgow, Glasgow, United Kingdom; 2 NHS Greater Glasgow and Clyde, Glasgow, United Kingdom; 3 Imperial College London, London, United Kingdom; 4 York and Scarborough Teaching Hospitals, York, United Kingdom,AFF>; 5 Newcastle University, Newcastle, United Kingdom; 6 North Cumbria Integrated Care NHS Foundation Trust, Carlisle, United Kingdom; 7 Luton and Dunstable Hospital, Luton, United Kingdom

## Abstract

Authors

Heather McAdam (presenting), Ruth Goh, Gloria Cheung, Felicity Allman, Samyak Pandey, Julia Alsop, Annabelle Hook, Jessica Randall, Benjamin Perry, Katherine Beck, David Codling, Judith R Harrison

**Aims:** Self-harm is increasingly prevalent among adolescents in the UK, with rising hospital admissions for those under 18. The updated National Institute for Health and Care Excellence (NICE) guidelines (NG225) for managing adolescent self-harm, published in September 2022, emphasised the need for timely, structured care, including risk assessments, psychosocial support, and family involvement. This study aimed to assess the clinical management of children and adolescents presenting to Emergency Departments (ED) for self-harm, evaluating compliance with the updated NICE guidelines across nine teaching hospitals in Scotland, England, and Wales.

**Methods:** This retrospective, multicentre study reviewed ED records of individuals aged 8–17 years who presented with self-harm between 7 September and 7 November 2022. Consecutive sampling was used, with data collected by medical student regional leads, who were recruited and trained through a national steering group. The leads followed a structured protocol to ensure consistency in reviewing records, focusing on risk assessments, psychosocial evaluations, consent for family involvement, and age-appropriate ward admissions. Data was centralised for analysis, where compliance with each audit criterion was assessed, and statistical analysis was conducted to identify trends and areas for improvement.

**Results:** A total of 328 patient records were analysed. The majority of patients were female (82.0%) and white (68.2%), with a mean age of 14.7 years (σ = 1.58). Compliance with NICE guidelines varied significantly across audit criteria. The highest compliance was for family involvement, with 73.5% of records documenting consent. However, social media interactions, a key component of risk assessment, were documented in only 21.5% of cases. Delayed psychosocial assessment was noted in 17.8% of records. Only 26.1% of 16–17-year-olds requiring inpatient care were admitted to age-appropriate wards, suggesting gaps in the provision of suitable care.

**Conclusion:** This audit demonstrates variability in adherence to the updated NICE guidelines across nine hospital sites. Family/carer involvement showed the highest compliance, but there were significant gaps in the use of risk assessment tools and timely psychosocial evaluations. The findings highlight the need for improvements in these areas and the importance of further training for clinical teams. The study also illustrates the value of student-led research in engaging future healthcare professionals in academic psychiatry and national data collection.

